# Supply chain resilience in the Colombian defense sector before and during the COVID-19 pandemic: A comparative study

**DOI:** 10.1371/journal.pone.0282793

**Published:** 2023-03-08

**Authors:** Benjamin Cabrera, Ricardo Santa, Thomas Tegethoff, Diego Morante, Mario Ferrer

**Affiliations:** 1 Universidad Icesi, Cali, Colombia; 2 Colegio de Estudios Superiores de Administración-CESA, Bogotá, Colombia; 3 Escuela Militar de Aviación “Marco Fidel Suárez”, Cali, Colombia; 4 Alfaisal University, Riyadh, Saudi Arabia; University of Malta, MALTA

## Abstract

Unforeseen events can significantly affect organizations’ supply chains and disrupt their continuous flow. Therefore, organizations need to develop a response capability that allows them to minimize the negative effect of such events and quickly recover from them, also known as resilience. This research performs a comparative analysis of the influence that risk, vulnerability, and adaptability have on the resilience capability of supply chains in Colombian defense sector organizations before and during the coronavirus outbreak. Based on a literature review, a survey was designed and applied online to collect data from respondents related to the activities of the Colombian Air Force supply chain. For the first wave, data was collected between December 2019 and January 2020. Data for the second wave was collected in August 2020. Results suggest that identifying and managing risks positively impact reducing vulnerability and increasing adaptability. Moreover, by decreasing exposure and improving adaptability, the organization positively influences supply chain resilience capability. The results also indicate that the pandemic positively affected risk and vulnerability awareness. The identification of vulnerabilities had a positive impact on the resilience capacity during the Corona Virus outbreak. This research provides relevant information for the Colombian government on developing public policies and mechanisms of service and support for defense sector organizations to strengthen their resilience capability. Likewise, the study offers valuable information to those organizations interested in improving their resilience capability and that of the sector in which they are involved.

## Introduction

This research began in mid-2019 by analyzing the supply chain in the defense sector in Colombia. Specifically, the vulnerability, the risk, and the adaptability influence the supply chain resilience capability. At the beginning of 2020, after analyzing the collected information, the World Health Organization declared that the coronavirus outbreak had become a pandemic on March 11^th^. This event impelled a reassessment of the research path. As a result, it was decided to collect new information using the same survey in the same sample to establish a comparison analyzing the same factors before the pandemic and during its influence.

Although the general concepts of supply chains and their management remain the same, the influence of the coronavirus pandemic has generated severe concerns in organizations regarding how supply chains have been managed until then. This forced them to rethink their structure and change their perspective regarding potential disruptions in their operation [1, 2].

According to the Council of Supply Chain Management Professionals (CSCMP), supply chain management (SCM) plans, coordinates, and executes all activities related to sourcing, converting, and logistics among its members, which may include external suppliers of materials and services, the manufacturer, commercial intermediaries, and end customers. SCM comprehensively manages supply and demand within and between companies to achieve a sustainable competitive advantage [3, 4]. All those activities require adequate coordination and integration to contribute to organizational performance. Organizations must be attentive to the changes that occur throughout the supply chain rather than individual processes or activities [5].

The concepts related to SCM apply to a multitude of organizations. However, there are significant differences in supply chain goals, strategy, and processes depending on the type of organization. Defense sector organizations manage a complex and unique supply chain due to the nature of their operations in national defense and other support activities.

Organizations in the private sector aim to maximize the total supply chain efficiency and operational effectiveness. Consequently, adequate supply chain management helps to compete for a higher market share and profit and, therefore, a sustainable competitive advantage [6, 7]. Governmental organizations, on the other hand, are looking for adequate benefits to the community, which does not necessarily include maximizing profit. In a national defense context, the ultimate goal is to optimize defense capability and provide material and resource readiness with an acceptable level of risk while minimizing the overall supply chain’s costs [5, 6].

Any disruption within the supply chain for a private company implies a loss of profit [8, 9], while any disturbance of the supply chain in the public sector implies a disruption of fundamental services for the community, i.e., the well-being of society [10]. Nevertheless, for the defense sector, the implications of a disruption in the supply chain on a battlefield, paying attention to an unusual event, or in an emergency is a matter of human lives, which deserves closer attention [11, 12].

An appropriate (continuous) flow of information, goods, and services within the supply chain is required to guarantee the efficiency of the processes. It should be considered one of the strategic goals of any organization, regardless of its type. Both public and private organizations are increasingly concerned about the level of risk and vulnerability caused by unexpected disruptions within their supply chain. This also affects how the organizations may develop strategies that allow them to reach adaptability and resilience [6, 13].

According to the Supply Chain Resilience Report [14], more than half of worldwide organizations experienced some significant supply chain disruption in 2019 (the year before the COVID-19 pandemic), mainly caused by IT outages (unplanned), adverse weather, loss of talent or skills, cyber-attack, transport network disruption, and political or regulatory changes. Supply disruptions were a significant reason for the closure of small and medium companies, revealing how severe the impact of these disruptions could be [15].

An adequate supply chain and logistics process work as a bridge between supplier locations (factories, warehouses, depots) and final users (markets, customers, citizens, warfighters), which are separated by time and distance. Those processes are fundamental to achieving organizational, operational effectiveness [16].

Because of market globalization, supply chain and logistic processes are becoming more complex, especially for Colombian organizations, which must deal with limited infrastructure, political unrest, security issues, and rugged topography. Additionally, several organizations with world-class supply chain practices compete in the same market. Consequently, Colombian organizations need to learn about the commercial conditions, logistics, and supply chain operations in the global and local markets. This helps improve their competitive position and resilience in the disruption of the supply chain [17].

Independent of how well-managed the supply chain is (coordinated, planned, implemented, and controlled), unexpected events may affect the process’s efficiency. The COVID-19 pandemic was an incredible event—considered the most severe global crisis since the second world war—and it is pretty sure that it will have a long-term impact on economies, supply chains, and trade relations. The different governmental containment measures have caused severe disruptions in the global supply chains [18], affecting the supply of components and raw materials manufactured in countries such as China, Vietnam, and India, that act as supply chain nodes because they hold massive and inexpensive materials and labor sources. This exposed vulnerabilities in numerous industries and sectors with no alternatives to switch rapidly to other sources [19]. This situation may encourage organizations to rethink their supply chain strategies by diversifying and having alternative suppliers [18].

Therefore, it should be an organizational priority to identify the factors that can affect its supply chain the most and try to prevent and handle potential disruptions. Although there are several studies in the literature analyzing factors that affect the supply chain, most of them have been carried out in developed countries, and few of them are focused on government institutions [16, 20–26]. Furthermore, there is no evidence of studies focused on Colombian defense organizations that consider the effect of risk, vulnerability, and adaptability on the supply chain’s resilience, which are the factors examined in this study. Therefore, the question addressed in this research is:

"What are the influence of risk, vulnerability, and adaptability on the supply chain resilience capability in organizations in the defense sector in Colombia before and during the coronavirus outbreak?"

To answer the research question, based on a review of the literature, a reliable instrument was designed to collect data from the Colombian Air Force, which has several facilities, bases of operations, and a vast network of suppliers. This study based its inferences on analyzing the interactions of the selected factors in the chosen institution at two different times: just before the coronavirus outbreak and during the pandemic. The Structural Equation Modeling (SEM) technique was used to analyze the collected information.

## Literature review

### Supply chain in the defense sector

The worldwide SCM’s best practices in the private sector have been adopted as a benchmark to assess the efficiency of organizations in other sectors in managing their supply chains. Nevertheless, using SCM best practices in public organizations is still incipient since few articles in the literature deal with SCM in the public sector [27–30]. It may be naïve (or irresponsible) to make a list of industries and choose a supply chain ’leader’ for all of them. Each industrial sector is unique and has its complexity when analyzing the supply chain [31]. Among those many sectors, defense is one of the most challenging.

For the defense sector, the mission of SCM must be to provide comprehensive logistical support for the different weapons and personnel. This support should be precise in its operation but minimize the cost of providing it [32]. Therefore, a relevant goal is to achieve an efficient but also effective supply chain through the continuous improvement of logistics processes. This may increase operational costs but also supports the readiness of weaponry within acceptable costs [5, 33]. This is a conflict–cost situation in a typical SCM trade-off decision.

Consequently, the defense sector has adopted business SCM practices, such as managing inventory levels [33], providing visibility and aligning appropriate information across the supply chain, improving cooperative and collaborative efforts to enhance the supply chain [31], and transferring some tasks to more efficient private companies via outsourcing [34]. These practices, besides reducing costs, may affect the risk level and vulnerability within the supply chain and even its adaptability and resilience capabilities.

However, "importing" those SCM best practices from the private to the defense sector is not easy, as there are differences between military logistics and supply chain and its private sector counterpart. These differences are striking in scope, scale, and environment. No private or public organization manages and controls a vast supply system and an assortment of supplies, equipment, and personnel as the defense forces. These supplies need to reach many combat "customers" (processing millions of requests) that may differ in their requirements and location [6, 35].

The scale of military logistic operations refers to the enormous quantity of items and weight that must be mobilized, far superior to any regular business operation [35]. Moreover, private businesses operate in a relatively predictable and generally peaceful environment. By contrast, military supply chains and logistics must operate in an environment that is often hostile, highly uncertain, volatile, complex, and ambiguous [6, 35].

Military logistics and supply chain operations are essential to any national armed force. The support during an ongoing deployment, the adequate response to any threats or natural disasters, and dealing with adverse conditions are critical in reaching operational efficiency. To accomplish these multiple purposes, the defense government institution must foster an improved, resilient, and adaptable supply chain by identifying its level of vulnerability and mitigating the supply chain risks to potential supply chain disruptions (terrorism, cyberattacks, unreliable suppliers, natural disasters, labor strikes), and trying to minimizing the cost and damages [33, 36].

A perfect example of an unexpected supply chain disruption is the COVID-19 pandemic that the world is experiencing, which has affected the supply chain of countless products and services in all sectors. For the defense sector, the adverse effects of this pandemic are evident in medical supplies [37], food [38], military equipment, and various products and services [39], not only due to the cascading effects derived from the economic crises that have affected supply chains by volatility in both supply and demand [40, 41] but also because countries were forced to reduce national defense budgets to address the social and public health effects of the pandemic [42, 43].

The COVID-19 outbreak has exposed several risks and vulnerabilities, which has compelled nations and organizations to develop adaptability and resilience in their supply chains [44, 45].

### Resilience

Disruptions in the supply chain have become a critical issue for nations and organizations in today’s unpredictable and fluctuating environment, especially troubled by the coronavirus pandemic. Global sourcing and outsourcing of relevant activities raise the risk of supply chain disruptions, making it challenging for managers to build resilience in their supply chains by identifying, managing, and mitigating such risks.

Although resilience is a multidisciplinary concept, in the Supply Chain Risk Management (SCRM) field, it is considered an adaptive organizational capacity that allows the supply chain to prepare, respond and recover from any interruption, often unexpected and in challenging conditions, maintaining operational control of its functions at an acceptable level [46, 47]. Following the disruption, resiliency also allows any network to return to its previous state or adapt to a new (and better) operating state [48, 49].

There is no specific blueprint in the literature on achieving resilience in the supply chain. However, five organizational capabilities are considered crucial in developing resilience:

1. Re-engineering: All supply chain members must understand their tasks and involvement and be aligned if a disruption could arise. Clearly defining the flow of the supply network implies understanding who its members are, what resources they have, what activities they carry out, and what control measures are implemented [47, 50, 51].
2. Collaboration: From a network perspective, supply chain collaboration brings the network together from a holistic view (as a whole), which is essential to build and strengthen its resilience capability, but it can only be achieved if members voluntarily share information, experience, and knowledge with each other [48, 50, 52].
3. Agility and adaptability: Agility comprises the flexibility and ability to respond adequately to environmental influences through the continuous search for a better response to change and uncertainty (short-term), facilitating the processes of coordination [50, 53, 54]. Adaptability is the organizational ability to restructure the supply chain, adjusting it to the environment over time by sensing market changes, threats, and opportunities (long-term) [55–57].
4. Risk awareness: There must be a clear and shared awareness among the members of the supply chain about their vulnerabilities and latent and potential risks so that, through joint support, they can evaluate them and join efforts to mitigate them [47, 50, 52].
5. Knowledge management practices: It is necessary to develop the ability to learn from past disruptive events to better prepare for future events, which can be achieved by sharing what is learned with customers, suppliers, and employees about risks in the supply chain, thus obtaining greater awareness and a better understanding of its structure and the information needed to act [47, 50, 58].

In pandemic times, supply chain resilience must focus on generating real-time adjustments while achieving proactive management in designing flexible redundancies that minimize external uncertainty in the supply networks [59]. Recovery and learning emerge as capacities that promote organizational resilience: the first allows resuming the activities before the interruption. In contrast, the second allows improving such activities as a result of what was learned during that interruption and its results [60].

The scope of supply chain resilience may be extended beyond survival by enabling intertwined supply networks to assimilate and endure an event as shocking as the COVID-19 pandemic [61, 62]. Other resilience strategies that may help to withstand such events are reallocating resources, restructuring the supply chain, developing more vital collaboration among its members [63], and increasing productivity by reducing costs through digitization (cloud-based services, AI, blockchain, big data) and automation, as resources allow [64], which may help to reduce both external and internal risks.

### Risk

The term "risk" describes unexpected events that affect the objectives. Within organizations, decision-makers associate risk with the threat of something that may happen and may disrupt the continuous flow of activities or prevent reaching the planned goals [65].

Risk refers to the chance (probabilistic measure) that a specific threat happens and the consequences (measure) of that event. Any event can occur inside (e.g., financial default of a supplier) or outside (e.g., an earthquake destroys production capacity) of the organization. Consequently, the organization must understand and manage the risk adequately and include in their business strategy their level of exposure to uncertainties and the potential consequences [65–67].

Although there is no unanimous consensus, five sources of risk related to the supply chain can be identified in the literature: environment, demand, supply, processes, and control. Environmental risks are those uncertainties that come from the political/legal, socio-economic, or natural environments that surround organizations. Demand risks are associated with the logistical flow of products to the market, while supply risks are associated with supplier relationships. Both (supply and demand) are generally internal to the chain. The design and implementation of flows and processes within and between supply chain members are considered another source of risk, as is the control of management agreements or policies established between such members. It is necessary to note that the sources mentioned can affect each other since environmental risks can lead to supply or demand risks in the chain, while the processes and control mechanisms, in turn, may amplify or absorb the effect of certain external or internal risks in the supply chain [68, 69].

The typhoon in Southeast Asia, the terrorist attacks in the USA in 2001, the SARS outbreak in 2002, the tsunami in Japan in 2011, and now the COVID-19 pandemic have revealed severe weaknesses, vulnerabilities, and risks in supply chains and their management [70, 71]. Consequently, risk management has become an essential factor in the organizational strategy and in designing SCRM processes, which implies a coordinated effort between the organizations involved to identify and manage risks, mitigate short-term and long-term vulnerabilities, and trace an operational effort toward the future [69]. Accordingly, the first hypothesis contends that risk identification has a predictive influence on vulnerability level (H1).

However, regardless of the risk management strategy, supply chain risks should be identified and managed holistically for an integrated supply chain [72]. In a permanently changing environment, SCRM may be an organizational ability to be flexible and adaptable [73]. Therefore, the second hypothesis asserts that risk identification has a predictive influence on organizational adaptability (H2).

Additionally, organizations must identify risks and respond appropriately to them, regardless of the risks’ sources. A better understanding of such sources not only improves the operational performance of the supply chain but can also help to better prepare it for disruptions by adjusting logistics and other activities [74]. Therefore, the third hypothesis in this research states that risk identification has a predictive influence on supply chain resilience capability (H3).

### Vulnerability

The term "risk" is also frequently used to describe something vulnerable or susceptible to damage [75]. Organizations must implement a proactive SCRM to identify their vulnerabilities since disruptions adversely affect supply chain outcomes (performance or financial) [76, 77]. The features of a supply chain are critical for determining its vulnerability and impact, including both the likelihood of and damages caused by disruptions [66].

Supply chain vulnerability includes the supply chain characteristics, variables, infrastructure, related processes, and environmental factors [78, 79]. Vulnerability is visible when multiple events present a risk to the supply chain that could cause unexpected interruptions in the flow of information, products, and knowledge along the chain [80]. The coronavirus pandemic had a profound impact on the global financial and economic order by demonstrating that global supply chains and their distribution structures are highly vulnerable to such disruptions, which will undoubtedly lead to a structural reconfiguration of previously globalized supply chains [81, 82].

Consequently, defense organizations must handle the supply chains’ vulnerabilities and risks to avoid potential interruptions and consequences, recognize the dynamic nature and extent of supply chain risk, and understand that supply chains are inter-organizational networks operating in an unpredictable environment. Nevertheless, it remains the managers’ obligation to identify, manage, and mitigate the effects of risks to reduce vulnerabilities in the supply chain [83] through developing organizational capabilities such as resilience and adaptability to face those uncontrollable forces. Accordingly, the following hypotheses argue that there is a predictive influence of vulnerability level on resilience capability (H4) and adaptability (H5).

### Adaptability

According to [84], an organization gains a competitive advantage when its supply chain is agile; it can react rapidly to unplanned changes in demand or supply, it can adapt over time as market composition changes, and it can align the members’ interests in the supply network, optimizing the supply chain’s performance and maximizing their interests. However, defense organizations do not require a competitive advantage but the ability to answer agilely to different challenges [85].

An organization develops adaptability when it has the skills to adapt to the environment to meet changing customer needs. The supply chain must evolve and change in tandem with the customers. As a result, supply chain adaptability trains its members to respond to evolving circumstances [84, 86], similar to the resilience concept discussed earlier. Accordingly, the following hypothesis proposes that there is a predictive influence of adaptability on resilience capability (H6).

However, it is necessary to establish the difference between agility and adaptability in an organizational context. The ability to adjust quickly to short-term market changes, such as variations in demand or supply patterns, shortages, or disruptions, is referred to as supply chain agility [84, 87]. The ability to rearrange the supply chain design and respond more radically to long-term developments is known as supply chain adaptability [55, 84]. The organizational capability to respond to changes can be characterized as a dynamic capability, a higher-order capability, which promotes the ability to detect opportunities and challenges and capitalize on them by shifting assets and organizational structures [56]. Adaptability is a dynamic transforming capability because the change in the supply chain structure is radical in the long term [55].

The COVID-19 outbreak exposed severe vulnerabilities in the supply chains of uncounted industries, including defense organizations, due to the use of the "benefits" that globalization allowed (before the pandemic) and the natural interdependence between the organizations involved, regardless of their geographic location [44, 82]. Part of the adjustments that are seen coming to the industry includes the fundamental restructuring of those global supply chains, which requires adaptability to mitigate threats and discover new opportunities in the long term [2, 81].

## Methodology

**Fig 1:**
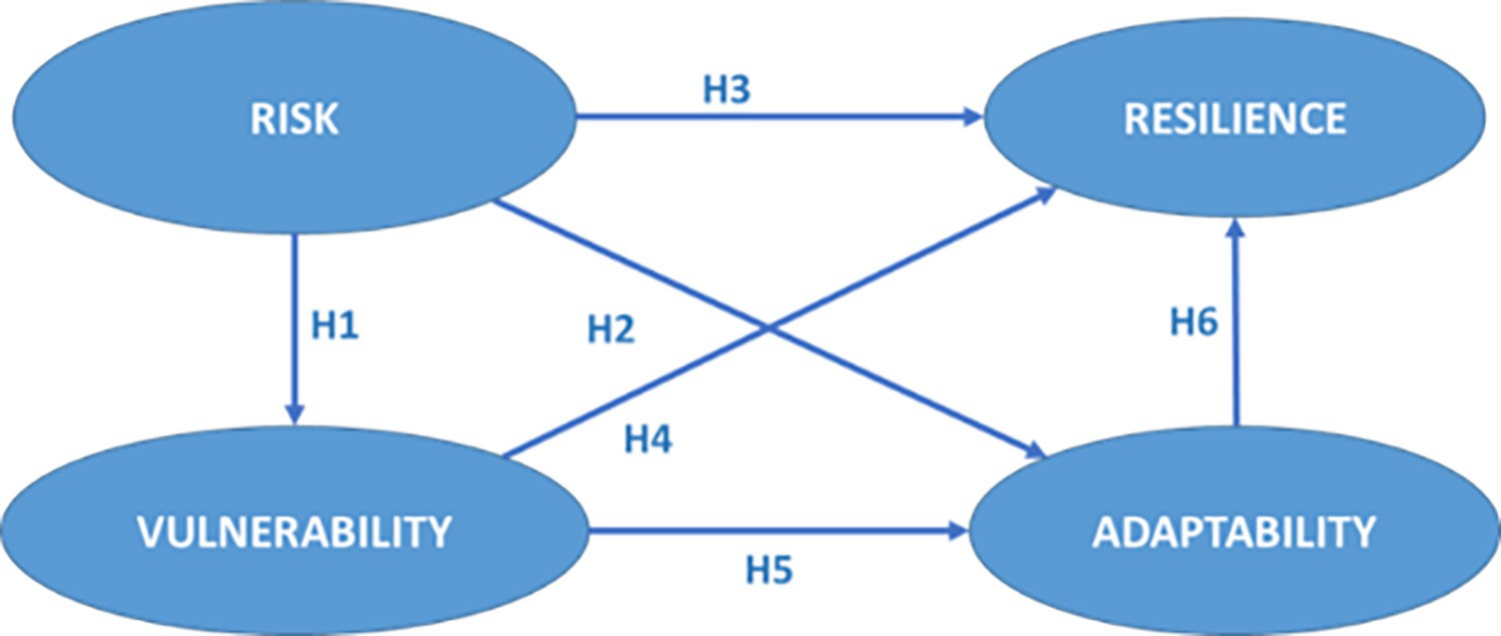
Supply chain resilience model. Source: By the authors.

Based on the literature review, the selected factors affect the supply chain resilience capability, but all the factors also affect each other. Thus, the primary goal of this study is to build on and expand existing research, as well as to present a theoretical model that analyzes the proposed hypothesis (see Fig 1):

The purpose of this research is confirmatory–correlational. A confirmatory analysis explains and quantifies the relationships between variables (Yin, 2013). Based on a thorough examination of the literature, a self-administered online questionnaire was constructed according to the parameters established by Hair et al. [88] and Evans and Mathur [89] guidelines. To avoid the social desirability bias, the questionnaire was reviewed by the ethics committee at the Colegio de Estudios Superiores de Administración (CESA) in Colombia to ensure that questions were neutral, unbiased, and non-threatening. Additionally, before filling out the questionnaire, the respondents were informed that all information provided would be treated in the strictest confidence, that the responses would be aggregated and used for research purposes, and that by completing this survey, they were giving their consent to such use, and that they might withdraw at any time. Consequently, consent was given by continuing with the survey, and at any point, the respondents had the possibility to close the online survey. The researchers followed ethical procedures during this research project by adherence to ethical standards.

The survey’s instrument was sent via email to Colombian Air Force employees from different areas linked to the institution’s supply chain, which has several facilities and a vast network of suppliers. This study based its inferences on analyzing the behaviors of the mentioned factors in the selected institution at two different times: Survey 1 was sent a few months before the coronavirus outbreak, and data collected was analyzed in January 2020, and Survey 2 was sent during the pandemic and analyzed in August 2020.

To assess the face and content validity, we extensively reviewed the literature to determine the relevant variables for the research. Subsequently, we performed a pilot with subject matter experts to define the final instrument. The surveys contain a demographic section, followed by a conceptualized set of variables to build a structural equation model for testing them using descriptive and inferential statistical analysis of the information collected. A Likert-type scale with five points was used to score statements relating to the operationalization of the model’s variables, with 1 representing strongly disagree and 5 meaning strongly agree. We added the survey as S1 Appendix. From Survey 1 (before the coronavirus outbreak), 120 questionnaires were returned that were considered valid and usable for the analysis, while from Survey 2 (during the pandemic), 216 were returned that were considered valid.

Information analysis was carried out using SEM. SEM is useful for testing hypotheses concerning relations between latent and observable variables by simultaneously estimating a set of multiple regression equations [90, 91]. Furthermore, SEM allows analysis of several dependent relations that capture the impacts of possible mediating constructs [91].

A confirmatory factor analysis (CFA) was performed to examine the relations between the latent and observable variables and determine the model’s robustness, validity, and reliability; a confirmatory factor analysis (CFA) was performed. To avoid cross-loadings and latent constructs being correlated, the load values (loading factors) were estimated, and it was validated that each item was used only in one construct. The Cronbach’s Alpha coefficients and the overall correlation of items were used to assess the model’s internal consistency [91, 92].

**Fig 2:**
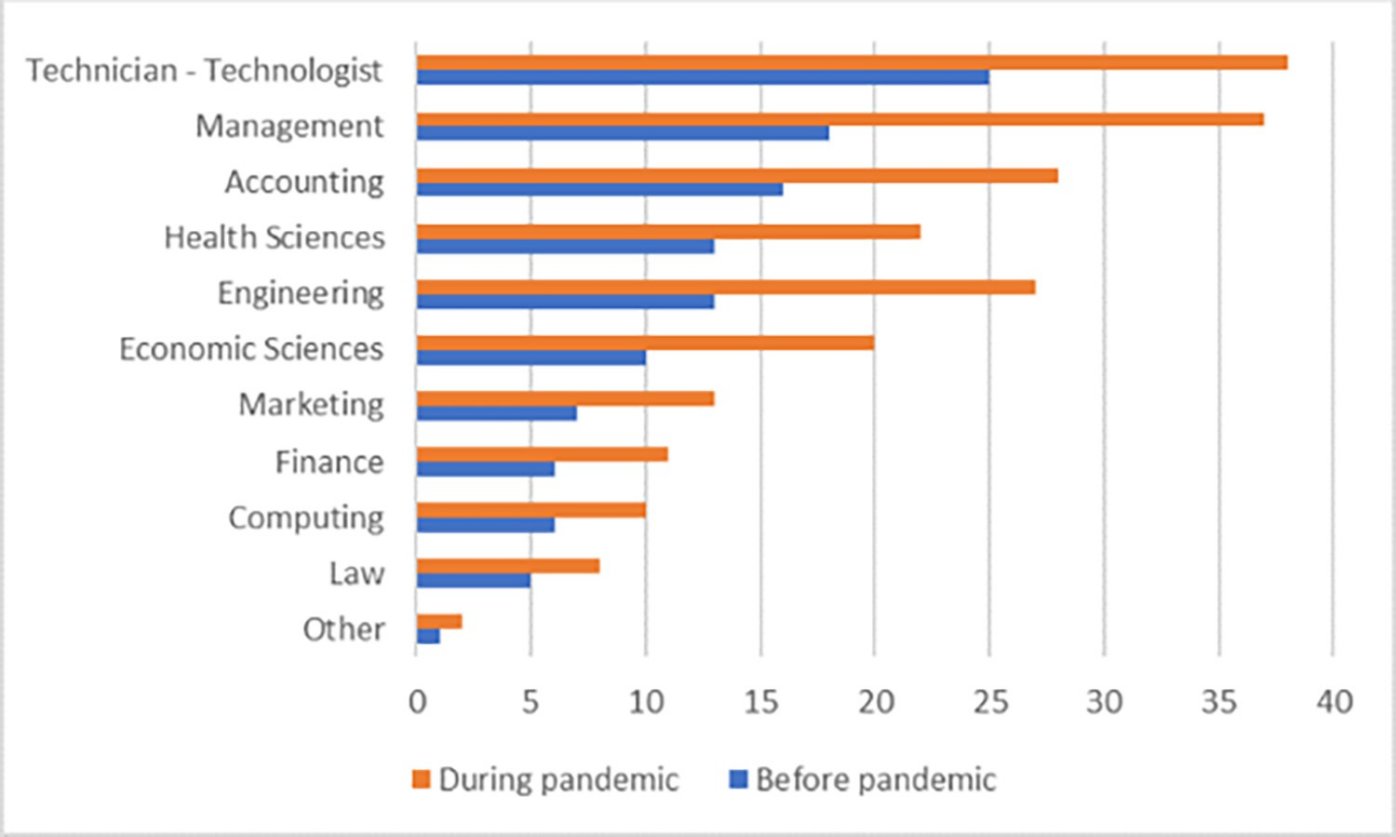
Professional education of the respondent. Source: By the authors.

Reviewing the demographic data, Fig 2 indicates the different educational backgrounds of the respondents, which shows a healthy diversity among them.

**Fig 3:**
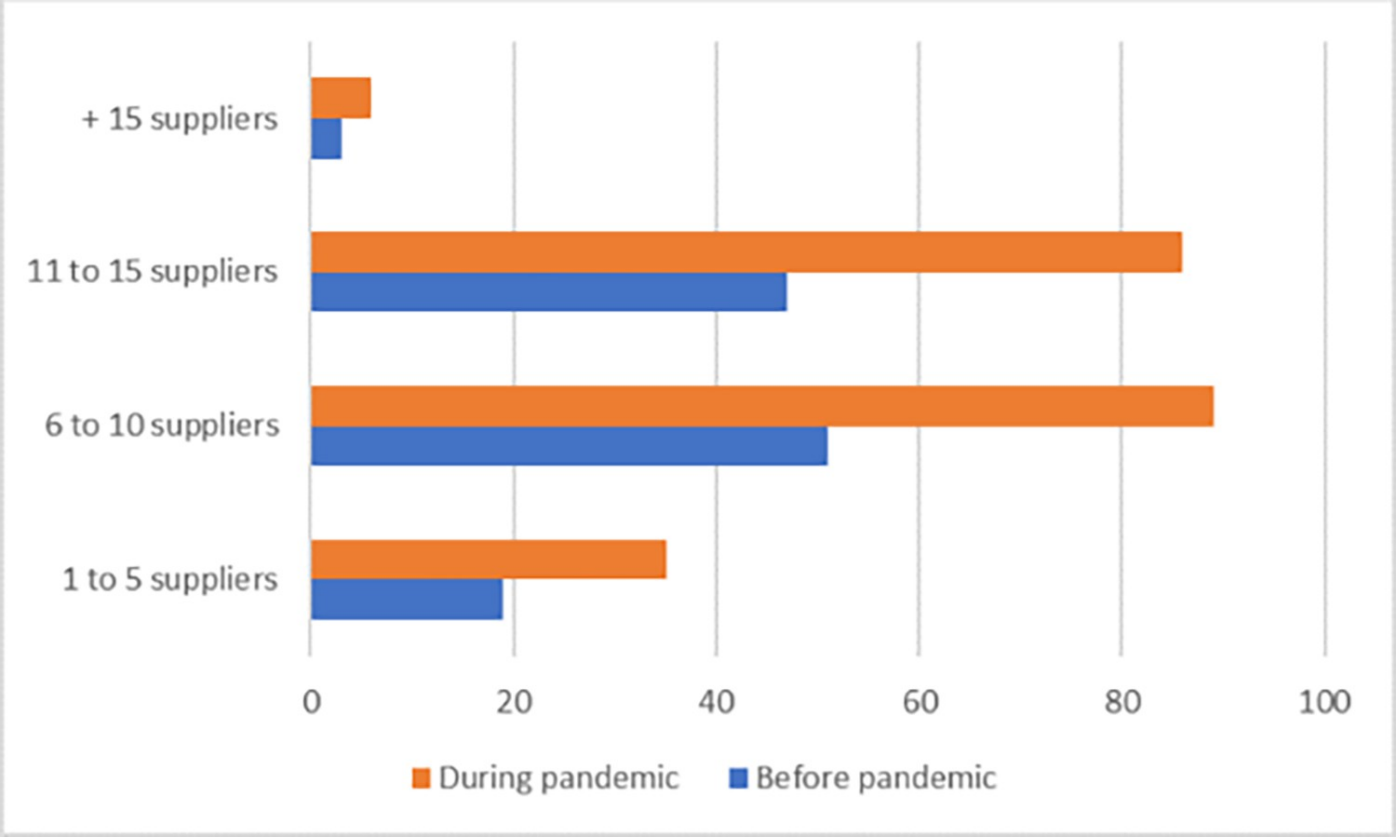
Number of suppliers. Source: By the authors.

**Fig 4:**
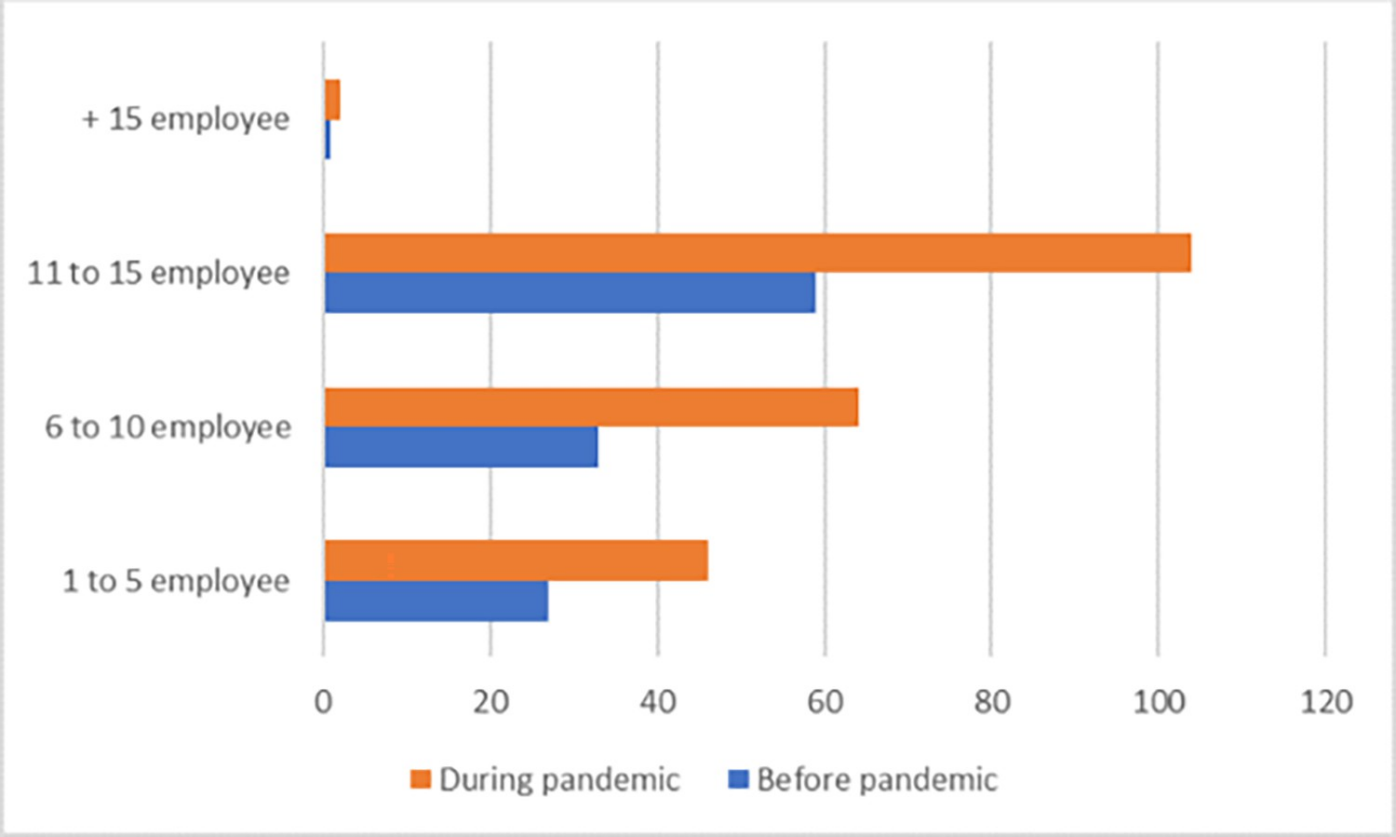
Employee related to supply chain. Source: By the authors.

Fig 3 shows the number of suppliers, companies, or third-party providers with whom there is a relationship in the respondent’s area of work. In contrast, Fig 4 shows the number of people working in the supply chain or related activities in the respondent’s work area. In both cases, none of them exceed 50%, which indicates the diversity of the sample.

**Fig 5:**
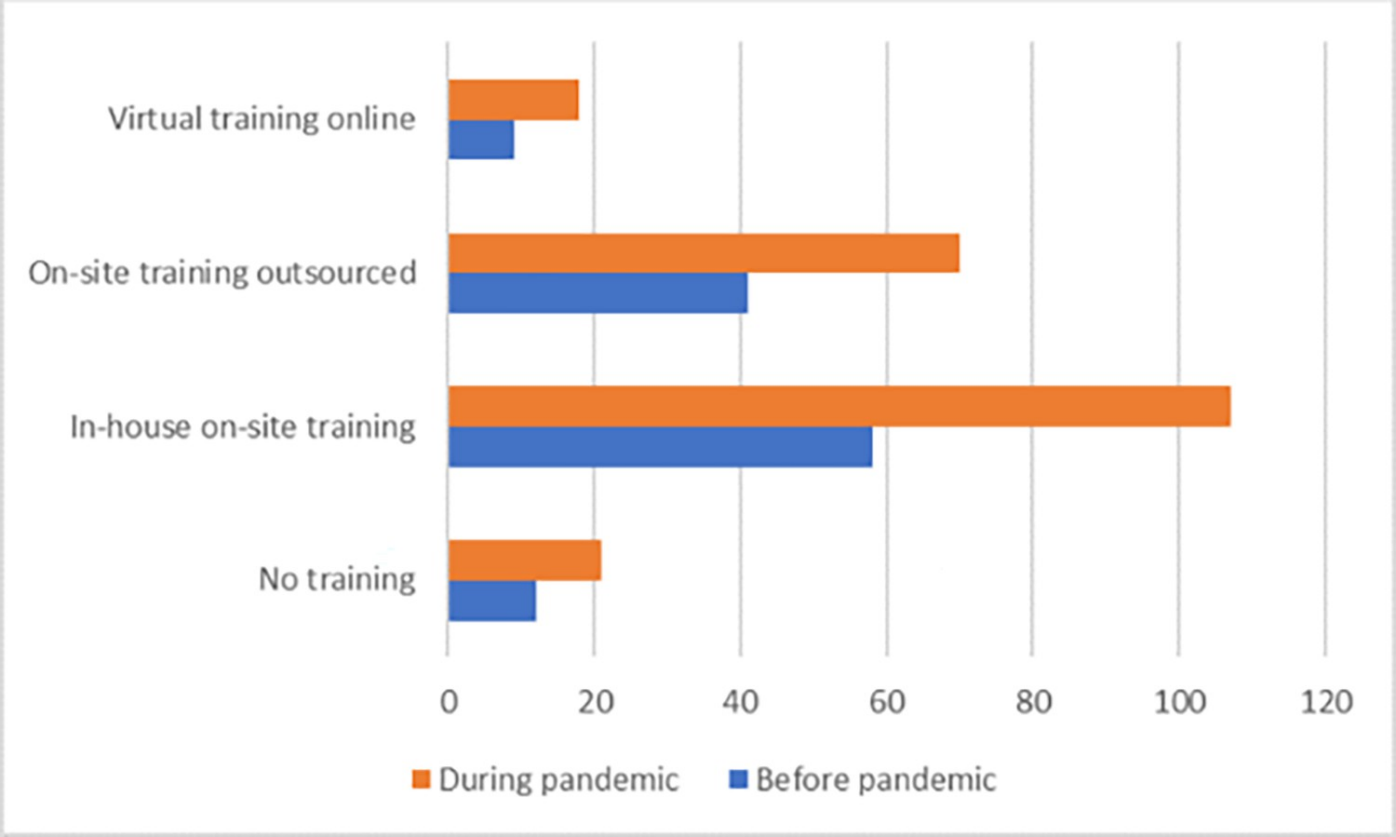
Risk prevention training. Source: By the authors.

Fig 5 shows that most of the respondents (around 90%) take some risk prevention training in their work area; however, the remaining portion does not receive any, which can be highlighted as an opportunity to improve and promote risk prevention training for all employees of the institution.

Table 1 shows the values of Cronbach’s Alpha coefficients of the constructs. All the coefficients have values above 0.7, established as the minimum acceptable cut-off level for confirmatory research [93–95].

**Table 1:** Cronbach’s Alpha coefficient.

	BEFORE COVID-19		DURING COVID-19	
Variable	Items	Cronbach’s Alpha	Items	Cronbach’s Alpha
Risk (Risk)	6	0.951	6	0.887
Vulnerability (Vulnerab)	4	0.894	7	0.857
Adaptability (Adaptab)	4	0.908	3	0.913
Resilience (Resilienc)	14	0.964	6	0.911

Source: By the authors

Table 2 shows the values of chi-square to degrees of freedom (CMIN/DF) of 1.695 and 1.915, which, being in a range between 1 and 5 [96] or in a range of 2:1 or 3:1 [97], can be taken as indicators of the robustness of the hypothesized model [98, 99].

**Table 2:** CMIN index.

	BEFORE COVID-19					DURING COVID-19				
Model	NPAR	CMIN	DF	P	CMIN/DF	NPAR	CMIN	DF	P	CMIN/DF
Default	52	379.593	224	.000	1.695	50	306.344	160	.000	1.915
Saturated	276	.000	0			210	.000	0		
Independence	23	2710.148	253	.000	10.712	20	3041.883	190	.000	16.010

Source: By the authors

Table 3 shows the robustness measure baseline comparisons, where all values are greater than 0.7 and close to or above 0.9, which are the recommended values [100], as well as the CFI (Comparative Fit Index) and GFI (Goodness-Of-Fit) in Table 4, also considered acceptable in each model since they are around the value of 0.9 [99, 101].

**Table 3:** Baseline comparisons.

	BEFORE COVID-19					DURING COVID-19				
Model	NFI Delta1	RFI rho1	IFI Delta2	TLI rho2	CFI	NFI Delta1	RFI rho1	IFI Delta2	TLI rho2	CFI
Default	.860	.842	.937	.928	.937	.899	.880	.949	.939	.949
Saturated	1.000		1.000		1.000	1.000		1.000		1.000
Independence	.000	.000	.000	.000	.000	.000	.000	.000	.000	.000

Source: By the authors

**Table 4:** RMR, GFI index.

	BEFORE COVID-19				DURING COVID-19			
Model	RMR	GFI	AGFI	PGFI	RMR	GFI	AGFI	PGFI
Default	.071	.886	.837	.638	.083	.876	.837	.667
Saturated	.000	1.000			.000	1.000		
Independence	.478	.218	.147	.200	.357	.288	.213	.261

Source: By the authors

The RMSEA (Root Mean Square Error of Approximation), which is acceptable as it is less than 0.08 [101–103], is shown in Table 5.

**Table 5:** RMSEA index.

	BEFORE COVID-19				DURING COVID-19			
Model	RMSEA	LO 90	HI 90	PCLOSE	RMSEA	LO 90	HI 90	PCLOSE
Default	.076	.063	.089	.001	.065	.054	.076	.013
Independence	.286	.276	.295	.000	.264	.256	.273	.000

Source: By the authors 1

The comparisons reviewed to measure robustness, reliability, and stability suggest that the model proposed is well represented by the variables and related data. Therefore, it is possible to make inferences based on these results.

## Compared results

**Fig 6:**
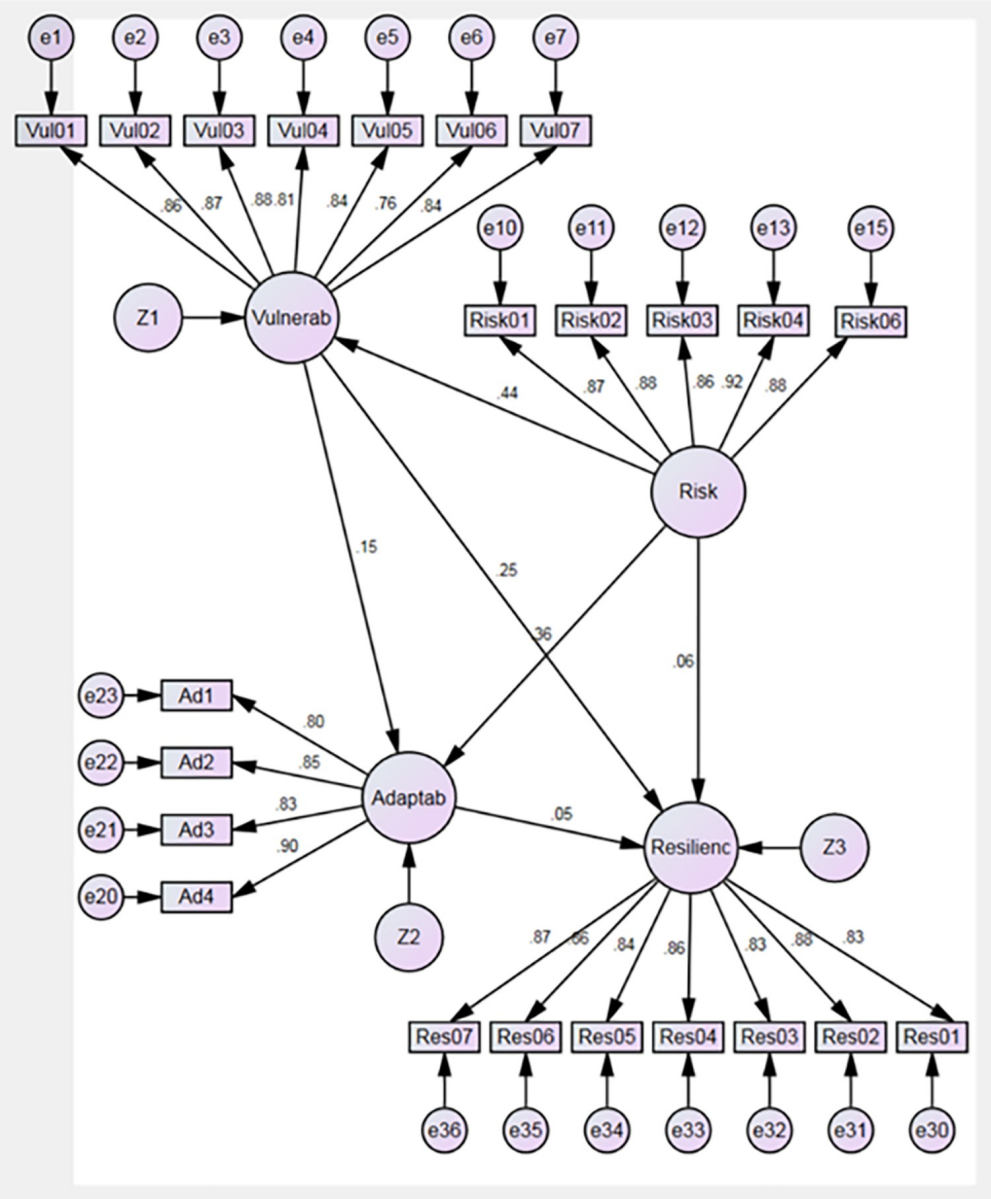
Result prior to pandemic. Source: By the authors 2.

**Fig 7:**
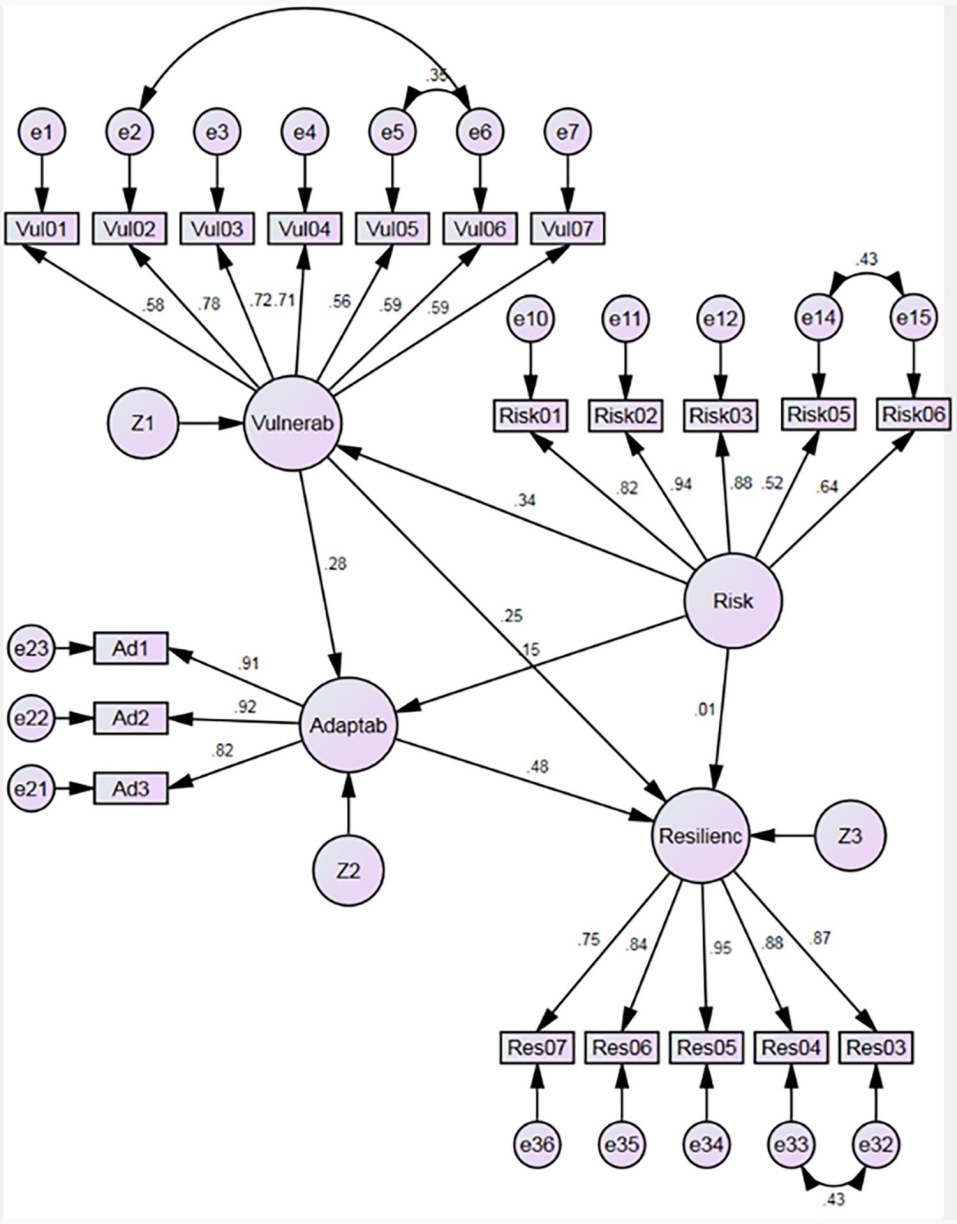
Results during pandemic. Source: By the authors 3.

The results shown in Table 6, and Figs 6 and 7 allow inferring that the identification and management of risk have a strong and positive impact on vulnerability reduction in the supply chain, which is supported by the theory [69], thus confirming hypothesis H1 both before and during the COVID-19 pandemic. At the same time, risk identification and management positively impact the organizational capability to be agile and adaptable [73], thus confirming hypothesis H2 before the pandemic and moderately confirming hypothesis H2 during the pandemic. It is likely that the extent of risk prevention training, even if it only reaches around 90%, indicates the people’s awareness to achieve this result [104, 105].

**Table 6:** Regression *w*eights (Default model).

			BEFORE COVID-19		DURING COVID-19	
			P	Label	P	Label
Risk	→	Vulner	***	H1-Confirmed	***	H1-Confirmed
Risk	→	Adapt	***	H2-Confirmed	.048	H2-Moderately confirmed
Risk	→	Resilie	.618	H3-No confirmed	.865	H3-No confirmed
Vulner	→	Resilie	.022	H4-Moderately confirmed	***	H4-Confirmed
Vulner	→	Adapt	.151	H5-No confirmed	***	H5-Confirmed
Adapt	→	Resilie	.636	H6-No confirmed	***	H6-Confirmed

Source: By the authors

However, the results suggest that identification and management of risk do not guarantee the development of resilience capacity in defense institutions since hypothesis H3 was not confirmed before or during the COVID-19 pandemic. This could be explained by theory since, in addition to adapting, resilience capacity implies that the supply chain prepares itself for unplanned events, responds to disruptions, and recovers from them by keeping running operations at an acceptable level of control [47], which is not so easy to achieve simply through training; it requires a more significant institutional effort and commitment [106, 107].

Results also show that hypothesis H4 was moderately confirmed before the COVID-19 pandemic and confirmed during the pandemic, suggesting that a decrease in vulnerability in the supply chain positively influences its resilience capability, which is supported by the literature [83].

An interesting result is shown when analyzing hypotheses H5 and H6 that were confirmed during the pandemic, which suggests that the decrease in vulnerability has a strong and positive influence on the improvement of adaptability, which, in turn, has a strong and positive influence on supply chain resilience acting as a moderating variable between vulnerability and resilience. Both results are supported by the literature [83, 84].

However, since hypotheses H5 and H6 were not confirmed before the pandemic, it may be suggested that adaptability, such as the organizational ability to radically restructure supply chain design to adjust to long-term market (environment) changes [55, 84], requires a significant institutional effort commitment [106, 107], and this ability to adapt was not a priority before the pandemic as the supply chain was operating well. It may be suggested that the significant impact caused by the pandemic on supply chains forced organizations to be aware of their risks and vulnerabilities [44, 45], forced them to adapt and, consequently, to develop their resilience capacity, in a much shorter term than usually is presumed to be reasonable [59, 62, 108].

## Discussion and conclusion

Christopher [109] noted that supply chain interdependency becomes more widespread when complexity and risk levels rise. Therefore, identifying and managing risks positively impact reducing vulnerability in the supply chain and its capability to adapt to unexpected events that may cause disruptions in its continuous flow. Most respondents had received some training in risk prevention, which undoubtedly contributed to this result. However, there is an opportunity to broaden the scope of risk identification and management training to the people across the entire institution, not only those involved in supply chain activities.

Five categories of supply chain risk sources have been identified in the literature: environment, demand, supply, process, and control [68, 69]. For specific risk sources affecting some level of the organization’s supply chain, adequate classification and identification are valuable to take more customized mitigation and control measures beyond taking general measures of prevention or reaction because simple identification and management of risks do not guarantee the development of resilience capacity in institutions, as the results showed. To this end, an institutional commitment is suggested that goes beyond adaptability capacity, supporting programs that enable the supply chain to prepare as well as possible, respond adequately, and recover quickly to sudden disruptions while retaining a desirable level of control and continuity of operations, which strengthens resilience.

As this document is being written, the world is facing one of the worst global crises of the last seven decades because of the COVID-19 outbreak, causing severe disruptions in the supply chains of several industries due to their global interconnectivity. The pandemic exposed vulnerabilities in supply chains that were previously considered adequate. Institutional commitment mentioned above must also accompany continuous improvement processes in building resilience capability involving the people in charge. Such processes must also be institutionalized so the same process feeds back to itself, understanding resilience as a capability to be developed constantly.

Finally, it is recommended that further studies be performed to validate the effects of the COVID-19 pandemic on the supply chain of institutions included in this study, to evaluate the level of preparedness, response, and recovery they possessed to face the event, to measure the damage caused, and, more importantly, to synthesize the lessons learned. This will assist in developing plans for containment and reaction to even less future severe events.

### Limitations

The research was conducted within the Colombian Air Force, part of the country’s defense organization. The Colombian Airforce is highly engineered; consequently, it remains to be seen if the results can be extended to other branches of the defense force, namely the Navy and Army.

## Supporting information

S1 Appendix (DOCX)

## References

[1] Shih W. Is it time to rethink globalized supply chains? MIT Sloan Management Review. 2020;61(4):1–3.

[2] Cordeiro MC, Santos L, Angelo ACM, Marujo LG. Research directions for supply chain management in facing pandemics: an assessment based on bibliometric analysis and systematic literature review. International Journal of Logistics Research and Applications. 2022;25(10):1313–33.

[3] Ballou R. Business logistics, supply chain management. Upper Saddle River, NJ: 5. internat. Aufl. Pearson Prentice Hall; 2004.

[4] Fan Y, Stevenson M. A review of supply chain risk management: definition, theory, and research agenda. International Journal of Physical Distribution & Logistics Management. 2018;48(3):205–30. doi: 10.1108/IJPDLM-01-2017-0043

[5] Peltz E, Robbins M, McGovern G. Integrating the department of defense supply chain. RAND NATIONAL DEFENSE RESEARCH INST SANTA MONICA CA, 2012.

[6] Haraburda S. Transforming military support processes from logistics to supply chain management. Army Sustainment. 2016;48(2):12–5.

[7] Storer M, Hyland P, Ferrer M, Santa R, Griffiths A. Strategic supply chain management factors influencing agribusiness innovation utilization. The International Journal of Logistics Management. 2014;25(3):487–521.

[8] Yu W, Jacobs MA, Chavez R, Yang J. Dynamism, disruption orientation, and resilience in the supply chain and the impacts on financial performance: A dynamic capabilities perspective. International Journal of Production Economics. 2019;218:352–62. doi: 10.1016/j.ijpe.2019.07.013

[9] Chen HL. Supply chain risk’s impact on corporate financial performance. International Journal of Operations & Production Management. 2018;38(3):713–31. doi: 10.1108/IJOPM-02-2016-0060

[10] Devi Y, Srivastava A, Koshta N, Chaudhuri A. The role of operations and supply chains in mitigating social disruptions caused by COVID-19: a stakeholder dynamic capabilities view. The International Journal of Logistics Management. 2021;ahead-of-print(ahead-of-print). doi: 10.1108/IJLM-04-2021-0235

[11] Gaustad G, Krystofik M, Bustamante M, Badami K. Circular economy strategies for mitigating critical material supply issues. Resources, Conservation and Recycling. 2018;135:24–33. doi: 10.1016/j.resconrec.2017.08.002

[12] Azadegan A, Dooley K. A Typology of Supply Network Resilience Strategies: Complex Collaborations in a Complex World. Journal of Supply Chain Management. 2021;57(1):17–26. doi: 10.1111/jscm.12256

[13] Stewart GT, Kolluru R, Smith M. Leveraging public‐private partnerships to improve community resilience in times of disaster. International Journal of Physical Distribution & Logistics Management. 2009;39(5):343–64.

[14] Elliott R, Thomas C, Muhammad K. Supply chain resilience report 2019. Business Continuity Institute. 2019.

[15] Raghuram P, Jawahar D. Empirical investigation of supply disruption in plastic SMEs. International Journal of Productivity and Quality Management. 2021;33(4):450–70.

[16] Ferrer M, Santa R. The mediating role of outsourcing in the relationship between speed, flexibility and performance: a Saudi Arabian study. International Journal of Productivity and Quality Management. 2017;22(3):395–412.

[17] Santa R, Ferrer M, Cabrera B, Castañeda C, Villano C. Does size matter? Impact of key supply chain competitive drivers on firm performance in the Valle del Cauca region in Colombia. EurOMA; 17.07–19.07; Helsinki—Finland2019.

[18] Stalenis G, Hodgson A, Boumphrey S. The impact of Coronavirus on the global economy. Passport, 2020.

[19] Nguyen MH, Phan AC, Matsui Y. Supply chain management in developing countries: empirical evidence from Vietnamese manufacturing companies. International Journal of Productivity and Quality Management. 2018;24(4):566–84.

[20] Babbar S, Addae H, Gosen J, Prasad S. Organizational factors affecting supply chains in developing countries. International Journal of Commerce and Management. 2008;18(3):234–51.

[21] Hao H-h, editor The key factors affecting supply chain risk towards emergencies. 2010 International Conference on Management and Service Science; 2010: IEEE.

[22] Hudnurkar M, Jakhar S, Rathod U. Factors affecting collaboration in supply chain: a literature review. Procedia-Social and Behavioral Sciences. 2014;133(1):189–202.

[23] Mwirigi N, Were S. Assessment of factors affecting supply chain management performance in Kenya public institutions-a case of the Judiciary. European Journal of Business Management. 2014;2(1):141–55.

[24] Ngoto AN, Kagiri A. Factors affecting supply chain management performance in international non-governmental organisations in Kenya. International Academic journal of procurement and supply chain management. 2016;2(1):37–49.

[25] Noor N, Saeed R, Lodhi RN. Factors affecting supply chain management effectiveness: A case of textile sector of Pakistan. Journal of Basic and Applied Scientific Research. 2013;3(11):56–63.

[26] Quesada H, Gazo R, Sanchez S. Critical factors affecting supply chain management: A case study in the US pallet industry. Pathways to supply chain excellence. 2012:33–56.

[27] Groznik A, Trkman P. Upstream supply chain management in e-government: The case of Slovenia. Government Information Quarterly. 2009;26(3):459–67.

[28] Nour MA, AbdelRahman AA, Fadlalla A. A context-based integrative framework for e-government initiatives. Government Information Quarterly. 2008;25(3):448–61.

[29] Burgess K, Singh PJ, Koroglu R. Supply chain management: a structured literature review and implications for future research. International Journal of Operations & Production Management. 2006;26(7):703–29.

[30] Yusuf A, Soediantono D. Supply Chain Management and Recommendations for Implementation in the Defense Industry: A Literature Review. International Journal of Social and Management Studies. 2022;3(3):63–77.

[31] Mayer A. Supply chain metrics that matter: A focus on aerospace & defense. Using financial data from corporate annual reports to better understand the aerospace & defense industry. Supply Chain Insights, LLC. 2014.

[32] Defense Logistics Agency. Fiscal Year (FY) 2020 President’s Budget. Operation and Maintenance, Defense-Wide. Department of Defense, 2019 March 2019. Report No.

[33] Haraburda S. Supply Chain Management: Maturity Level Assessment. Defense Acquisition Research Journal: A Publication of the Defense Acquisition University. 2017;24(4).

[34] Glas A, Hofmann E, Eßig M. Performance-based logistics: a portfolio for contracting military supply. International Journal of Physical Distribution & Logistics Management. 2013;43(2):97–115.

[35] Kress M. Operational logistics. 2 ed. Switzerland: Springer International Publishing; 2016.

[36] Zeimpekis V, Kaimakamis G, Daras NJ. Military Logistics: Research Advances and Future Trends: Springer; 2014.

[37] Pasquier P, Luft A, Gillard J, Boutonnet M, Vallet C, Pontier J, et al. How do we fight COVID-19? Military medical actions in the war against the COVID-19 pandemic in France. British Medical Journal Publishing Group; 2021. p. 269–74. doi: 10.1136/bmjmilitary-2020-001569 32759228

[38] Wincewicz-Bosy M, Sadowski A, Wąsowska K, Galar Z, Dymyt M. Military Food Supply Chain during the COVID-19 Pandemic. Sustainability. 2022;14(4):2464.

[39] Melnyk SA, Schoenherr T, Verter V, Evans C, Shanley C. The pandemic and SME supply chains: Learning from early experiences of SME suppliers in the US defense industry. Journal of Purchasing and Supply Management. 2021;27(4):100714.

[40] Brodeur A, Gray D, Islam A, Bhuiyan S. A literature review of the economics of COVID‐19. Journal of Economic Surveys. 2021;35(4):1007–44. doi: 10.1111/joes.12423 34230772 PMC8250825

[41] Araz OM, Choi T-M, Olson D, Salman FS. Data analytics for operational risk management. Decision Sciences. 2020;51(6):1316–9.

[42] Farias HC. Geopolitics and National Defense Capabilities: a look at the emerging scenario in pandemic times. 2020.

[43] Skliar N, Begma V, Vrublevska O. New challenges for the defense industrial enterprises of Ukraine in the conditions of the global pandemic. Amazonia Investiga. 2021;10(37):45–55.

[44] Chenarides L, Manfredo M, Richards TJ. COVID‐19 and food supply chains. Applied Economic Perspectives and Policy. 2021;43(1):270–9.

[45] Dyatkin B. COVID-19 pandemic highlights need for US policies that increase supply chain resilience. Mrs Bulletin. 2020;45(10):794–6. doi: 10.1557/mrs.2020.258 33437116 PMC7790014

[46] Weick K, Sutcliffe K, Obstfeld D. Organizing for high reliability: Processes of collective mindfulness. Crisis management. 2008;3(1):81–123.

[47] Ponomarov SY, Holcomb MC. Understanding the concept of supply chain resilience. The international journal of logistics management. 2009;20(1):124–43.

[48] Christopher M, Peck H. Building the resilient supply chain. International Journal of Logistics Management. 2004;15(2):1–13.

[49] Lengnick-Hall CA, Beck TE, Lengnick-Hall ML. Developing a capacity for organizational resilience through strategic human resource management. Human Resource Management Review. 2011;21(3):243–55.

[50] Scholten K, Scott PS, Fynes B. Mitigation processes–antecedents for building supply chain resilience. Supply Chain Management: An International Journal. 2014;19(2):211–28.

[51] Sheffi Y. Supply chain management under the threat of international terrorism. The International Journal of logistics management. 2001;12(2):1–11.

[52] Faisal MN, Banwet DK, Shankar R. Supply chain risk mitigation: modeling the enablers. Business Process Management Journal. 2006;12(4):535–52.

[53] Jüttner U, Maklan S. Supply chain resilience in the global financial crisis: an empirical study. Supply Chain Management: An International Journal. 2011;16(4):246–59.

[54] Saleeshya P, Sachin B. Modelling and analysis of an agile supply chain. International Journal of Productivity and Quality Management. 2015;15(4):486–510.

[55] Eckstein D, Goellner M, Blome C, Henke M. The performance impact of supply chain agility and supply chain adaptability: the moderating effect of product complexity. International Journal of Production Research. 2015;53(10):3028–46.

[56] Teece DJ. Explicating dynamic capabilities: the nature and microfoundations of (sustainable) enterprise performance. Strategic management journal. 2007;28(13):1319–50.

[57] Pool JK, Jamkhaneh HB, Tabaeeian RA, Tavakoli H, Shahin A. The effect of business intelligence adoption on agile supply chain performance. International Journal of Productivity and Quality Management. 2018;23(3):289–306.

[58] Blackhurst J, Dunn KS, Craighead CW. An empirically derived framework of global supply resiliency. Journal of business logistics. 2011;32(4):374–91.

[59] Ivanov D, Das A. Coronavirus (COVID-19/SARS-CoV-2) and supply chain resilience: A research note. International Journal of Integrated Supply Management. 2020;13(1):90–102.

[60] de Sousa Jabbour ABL, Jabbour CJC, Hingley M, Vilalta-Perdomo EL, Ramsden G, Twigg D. Sustainability of supply chains in the wake of the coronavirus (COVID-19/SARS-CoV-2) pandemic: lessons and trends. Modern supply chain research and applications. 2020;2(3):117–22.

[61] Nasereldin YA, Brenya R, Bassey AP, Ibrahim IE, Alnadari F, Nasiru MM, et al. Is the global food supply chain during the COVID-19 pandemic resilient? a review paper. Open Journal of Business and Management. 2020;9(1):184–95.

[62] Ivanov D, Dolgui A. Viability of intertwined supply networks: extending the supply chain resilience angles towards survivability. A position paper motivated by COVID-19 outbreak. International Journal of Production Research. 2020;58(10):2904–15.

[63] Chowdhury P, Paul SK, Kaisar S, Moktadir MA. COVID-19 pandemic related supply chain studies: A systematic review. Transportation Research Part E: Logistics and Transportation Review. 2021;148:102271. doi: 10.1016/j.tre.2021.102271 33613082 PMC7881707

[64] Burgos D, Ivanov D. Food retail supply chain resilience and the COVID-19 pandemic: A digital twin-based impact analysis and improvement directions. Transportation Research Part E: Logistics and Transportation Review. 2021;152:102412. doi: 10.1016/j.tre.2021.102412 34934397 PMC8677600

[65] DeLoach JW. Enterprise-wide risk management: strategies for linking risk and opportunity: Financial Times Prentice Hall; 2000.

[66] Wagner SM, Bode C. An empirical examination of supply chain performance along several dimensions of risk. Journal of business logistics. 2008;29(1):307–25.

[67] Pidgeon N, Hood C, Jones D, Turner B, Gibson R. Risk: analysis, perception, management Chapter 5 risk perception Report of a royal society study group. London, England7 The Royal Society. 1992.

[68] Mason-Jones R, Towill DR. Shrinking the supply chain uncertainty circle. IOM control. 1998;24(7):17–22.

[69] Jüttner U. Supply chain risk management. Understanding the business requirements from a practitioner perspective. The international journal of logistics management. 2005;16(1):120–41.

[70] Ivanov D. Predicting the impacts of epidemic outbreaks on global supply chains: A simulation-based analysis on the coronavirus outbreak (COVID-19/SARS-CoV-2) case. Transportation Research Part E: Logistics and Transportation Review. 2020;136:101922. doi: 10.1016/j.tre.2020.101922 32288597 PMC7147532

[71] Yang J, Xie H, Yu G, Liu M. Antecedents and consequences of supply chain risk management capabilities: An investigation in the post-coronavirus crisis. International Journal of Production Research. 2021;59(5):1573–85.

[72] Rao S, Goldsby TJ. Supply chain risks: a review and typology. The International Journal of Logistics Management. 2009;20(1):97–123.

[73] Lavastre O, Gunasekaran A, Spalanzani A. Supply chain risk management in French companies. Decision Support Systems. 2012;52(4):828–38.

[74] Chand M, Raj T, Shankar R. Risk mitigations strategy in supply chain planning and control: an ANP approach. International Journal of Productivity and Quality Management. 2015;16(1):92–113.

[75] Butterfield JE. Collins English Dictionary. Glasgow: HarperCollins; 2000.

[76] Christopher M, Lee H. Mitigating supply chain risk through improved confidence. International journal of physical distribution & logistics management. 2004;34(5):388–96.

[77] Lockamy A III, McCormack K. Analysing risks in supply networks to facilitate outsourcing decisions. International Journal of Production Research. 2010;48(2):593–611.

[78] Asbjørnslett BE. Assessing the vulnerability of supply chains. Supply chain risk: A handbook of assessment, management, and performance. 2009:15–33.

[79] Bakshi N, Kleindorfer P. Co‐opetition and investment for supply‐chain resilience. Production and Operations Management. 2009;18(6):583–603.

[80] Ferrer M, Santa R, Almadani SA. The interplay between competitive drivers, outsourcing and supply chain performance: the case of middle east supply chains. International Journal of Accounting Information Science & Leadership. 2013;6(17):107–17.

[81] Allen J, Burns N, Garrett L, Haass RN, Ikenberry GJ, Mahbubani K, et al. How the world will look after the coronavirus pandemic. Foreign Policy. 2020;20(2020):97–103.

[82] Xu Z, Elomri A, Kerbache L, El Omri A. Impacts of COVID-19 on global supply chains: facts and perspectives. IEEE Engineering Management Review. 2020;48(3):153–66.

[83] Peck H. Drivers of supply chain vulnerability: an integrated framework. International journal of physical distribution & logistics management. 2005;35(4):210–32.

[84] Lee HL. The triple-A supply chain. Harvard business review. 2004;82(10):102–13. 15559579

[85] Gholami M, Elahifard E, Rahmdel M, Iranshahi H. Factors Affecting the Agility of Defense Logistics. Innovation Management in Defensive Organizations. 2021;4(2):23–48.

[86] Dubey R, Altay N, Gunasekaran A, Blome C, Papadopoulos T, Childe SJ. Supply Chain Agility, Adaptability and Alignment: Empirical Evidence from the Indian Auto Components Industry. International Journal of Operations & Production Management. 2017;38(1):129–48.

[87] Blome C, Schoenherr T, Rexhausen D. Antecedents and enablers of supply chain agility and its effect on performance: a dynamic capabilities perspective. International Journal of Production Research. 2013;51(4):1295–318.

[88] Hair JF, Black WC, Babin BJ. Multivariate Data Analysis: A Global Perspective: Pearson Education; 2010.

[89] Evans JR, Mathur A. The value of online surveys: A look back and a look ahead. Internet Research. 2018.

[90] Hoyle RH. Structural equation modeling: Concepts, issues, and applications: Sage; 1995.

[91] Hair J, Black W, Babin B, Anderson R. Multivariate data analysis: a global perspective. 7 ed: Pearson Education; 2010.

[92] Cooksey RW. Illustrating statistical procedures: For business, behavioural & social science research: Tilde University Press; 2007.

[93] Nunnally J, Bernstein I. Psychometric Theory 3rd edition (MacGraw-Hill, New York). 1994.

[94] Bujang MA, Omar ED, Baharum NA. A Review on Sample Size Determination for Cronbach’s Alpha Test: A Simple Guide for Researchers. The Malaysian journal of medical sciences: MJMS. 2018;25(6):85–99. Epub 2018/12/28. doi: 10.21315/mjms2018.25.6.9 . 30914882 PMC6422571

[95] Taber KS. The Use of Cronbach’s Alpha When Developing and Reporting Research Instruments in Science Education. Research in Science Education. 2017;48(6):1273–96. doi: 10.1007/s11165-016-9602-2

[96] Wheaton B, Muthen B, Alwin DF, Summers GF. Assessing reliability and stability in panel models. Sociological methodology. 1977;8:84–136.

[97] McIver J, Carmines EG. Unidimensional scaling: Sage; 1981.

[98] Shi D, Maydeu-Olivares A. The effect of estimation methods on SEM fit indices. Educational and Psychological Measurement. 2020;80(3):421–45. doi: 10.1177/0013164419885164 32425213 PMC7221491

[99] Shi D, Lee T, Maydeu-Olivares A. Understanding the model size effect on SEM fit indices. Educational and psychological measurement. 2019;79(2):310–34. doi: 10.1177/0013164418783530 30911195 PMC6425088

[100] Ho R. Handbook of univariate and multivariate data analysis and interpretation with SPSS: Chapman and Hall/CRC; 2006.

[101] Bentler PM. Comparative fit indexes in structural models. Psychological bulletin. 1990;107(2):238. doi: 10.1037/0033-2909.107.2.238 2320703

[102] Jöreskog KG, Sörbom D. Recent developments in structural equation modeling. Journal of marketing research. 1982;19(4):404–16.

[103] Xia Y, Yang Y. RMSEA, CFI, and TLI in structural equation modeling with ordered categorical data: The story they tell depends on the estimation methods. Behavior research methods. 2019;51(1):409–28. doi: 10.3758/s13428-018-1055-2 29869222

[104] Norrman A, Wieland A. The development of supply chain risk management over time: revisiting Ericsson. International Journal of Physical Distribution & Logistics Management. 2020;50(6):641–66.

[105] Rogers H, Srivastava M, Pawar KS, Shah J. Supply chain risk management in India–practical insights. International Journal of Logistics Research and Applications. 2016;19(4):278–99.

[106] Ishida S. Perspectives on supply chain management in a pandemic and the post-COVID-19 era. IEEE Engineering Management Review. 2020;48(3):146–52.

[107] Moosavi J, Fathollahi-Fard AM, Dulebenets MA. Supply chain disruption during the COVID-19 pandemic: Recognizing potential disruption management strategies. International Journal of Disaster Risk Reduction. 2022:102983. doi: 10.1016/j.ijdrr.2022.102983 35475018 PMC9027543

[108] Aldrighetti R, Battini D, Ivanov D, Zennaro I. Costs of resilience and disruptions in supply chain network design models: a review and future research directions. International Journal of Production Economics. 2021;235:108103.

[109] Christopher M. Logistics & supply chain management: Pearson Uk; 2016.

