# Supplementary material for: Supply chain resilience in the Colombian defense sector before and during the COVID-19 pandemic: A comparative study
Source: PLoS One. 2023 Mar 8;18(3):e0282793. doi: 10.1371/journal.pone.0282793 (PMC9994741; doi:10.1371/journal.pone.0282793)
Supplement: S1 Appendix — (DOCX) [file pone.0282793.s001.docx]

**Appendix A**

**Assessment of the Colombian Air Force's risk management practices, vulnerability, adaptability and resilience in the supply chain**

**Definition of Vulnerability: “**An exposure to serious disturbance, arising from risks within the supply chain as well as risks external to the supply chain”.

| **Vulnerability** | | | | | |
| --- | --- | --- | --- | --- | --- |
| **Mark on a scale of 1 to 5, knowing that 1 indicates that you strongly disagree and 5 indicates that you completely agree, how relevant the following statements are to you:**  In his opinion, the following developments **contribute to an increase in vulnerability** within the supply chain: | **Strongly disagree** | **Disagree** | **Neither agree nor disagree** | **I agree** | **Completely agree** |
|  | **1** | **2** | **3** | **4** | **5** |
| Focus on efficiency instead of security aspects |  |  |  |  |  |
| Globalization of the supply chain |  |  |  |  |  |
| Focus on centralized distribution. |  |  |  |  |  |
| Enforced outsourcing |  |  |  |  |  |
| Reduction of suppliers |  |  |  |  |  |
| Single sourcing |  |  |  |  |  |
| Increased product variety |  |  |  |  |  |
| Centralized production |  |  |  |  |  |
| Uncontrolled suppliers lead times |  |  |  |  |  |
| Limited transportation infrastructure |  |  |  |  |  |
| Single financial source |  |  |  |  |  |
| Limited communication protocols |  |  |  |  |  |
| Long cash-to-cash cycle |  |  |  |  |  |
| In general, my company considers our supply chain significantly vulnerable to incidences |  |  |  |  |  |

| **Process Vulnerability** | | | | | |
| --- | --- | --- | --- | --- | --- |
| On a scale from 1 to 5, where 1 is strongly disagree and 5 is strongly agree, please rate the following statements.  Consider your relationships with both suppliers and customer.  Please, rate your level of agreement on the following risk related statements: | **Strongly disagree** | **Disagree** | **Ni de acuerdo ni en desacuerdo** | **I agree** | **Completely agree** |
|  | **1** | **2** | **3** | **4** | **5** |
| Our products/services face unpredictable demand changes from the customer or user |  |  |  |  |  |
| Raw materials for our product/design are scarce or in high demand. |  |  |  |  |  |
| The availability of our utilities (electricity, water, sewage) for production is poor. |  |  |  |  |  |
| Some equipment/products used in our operations are prone to failure. |  |  |  |  |  |
| Our production capacity is limited. |  |  |  |  |  |
| We have limited access to the ability to distribute products/services. |  |  |  |  |  |
| **Vendor vulnerability** | | | | | |
| Our suppliers frequently face significant disruptions. |  |  |  |  |  |
| Our suppliers have limited ability to cope with unplanned changes in demand. |  |  |  |  |  |
| We often face the loss of key suppliers during operations |  |  |  |  |  |
| Our customers frequently face significant disruptions. |  |  |  |  |  |
| **Technological vulnerability** | | | | | |
| Technological changes in our industry greatly affect the design and performance of our services/products. |  |  |  |  |  |
| We regularly face unforeseen technological failures in our operations. |  |  |  |  |  |
| It is difficult to adapt new technologies to our processes. |  |  |  |  |  |
| We are able to develop new technologies. |  |  |  |  |  |

| **Environmental vulnerability** | | | | | |
| --- | --- | --- | --- | --- | --- |
| Social and cultural changes have had a significant impact on our ability to provide our products or services. |  |  |  |  |  |
| Our facilities/operations are frequently exposed to adverse weather events or natural disasters. |  |  |  |  |  |
| **Physical vulnerability** | | | | | |
| Our products are regularly stolen or vandalized. |  |  |  |  |  |
| We often face accidents during operations/productions (i.e. fires, worker accidents)). |  |  |  |  |  |
| Our facilities or personnel may be the target of terrorism or sabotage. |  |  |  |  |  |
| **Financial vulnerability** | | | | | |
| Changes in financial and economic policies greatly affect our money and asset management. |  |  |  |  |  |
| We lack financial resources to cover all potential needs. |  |  |  |  |  |

**Definition of Risk:** “Anything that [disrupts or impedes] the information, material or product flows from original suppliers to the delivery of the final product to the ultimate end- user”.

| **Risk** | | | | | |
| --- | --- | --- | --- | --- | --- |
| On a scale from 1 to 5, where 1 is strongly disagree and 5 is strongly agree, please rate the following statements.  Consider your relationships with both suppliers and customer.  Please, rate your level of agreement on the following risk related statements: | **Strongly disagree** | **Disagree** | **Neither agree nor disagree** | **I agree** | **Completely agree** |
|  | **1** | **2** | **3** | **4** | **5** |
| There is a process to identify risks events in our supply chain |  |  |  |  |  |
| We work jointly with our partners to asses nature and extent of potential risk events |  |  |  |  |  |
| Risk events monitoring is a common practice in our supply chain |  |  |  |  |  |
| Risk events monitoring information is widely shared among our supply chain partners |  |  |  |  |  |
| Significant knowledge is built through the process of monitoring and addressing risk events |  |  |  |  |  |
| There is a clear policy regarding sharing losses after risk events in our supply chain |  |  |  |  |  |
|  | | | | | |
| **The following statements relate the estimation of the probability of occurrence and the consequences of the following risks concerning your company. Please, consider each statement carefully and then indicate your level of agreement.** | | | | | |
| Raw material supplier failure |  |  |  |  |  |
| Supplier quality problems |  |  |  |  |  |
| Finished goods manufacturing failure |  |  |  |  |  |
| Oil price crisis |  |  |  |  |  |
| Terrorist attack |  |  |  |  |  |
| Strike |  |  |  |  |  |
| Malfunction of the IT-system |  |  |  |  |  |
| Accident (e.g. fire) |  |  |  |  |  |
| Natural disaster |  |  |  |  |  |
| Machine breakdowns |  |  |  |  |  |
| Import or export restrictions |  |  |  |  |  |
| Transportation carrier failure |  |  |  |  |  |
| Transportation infrastructure collapse |  |  |  |  |  |
| Delivery chain disruptions |  |  |  |  |  |
| Sudden increase of custom duty |  |  |  |  |  |
| Change in customer demand |  |  |  |  |  |
| Technological change |  |  |  |  |  |
| Spike in raw material costs |  |  |  |  |  |
| Political boundaries |  |  |  |  |  |
| Cash crisis due to customers delaying payment |  |  |  |  |  |
| Price collapse due to a new competitor |  |  |  |  |  |
| Security violations and executive misdeeds |  |  |  |  |  |
| Extended loss of electricity (>1 day) |  |  |  |  |  |

| **Adaptability to confront risk** | | | | | |
| --- | --- | --- | --- | --- | --- |
| We realign our resources and processes in response to environmental changes. |  |  |  |  |  |
| We reconfigure our resources and processes in response to the dynamic environment. |  |  |  |  |  |
| We restructure our resource base (raw materials) to react to the changing business environment. |  |  |  |  |  |
| Revamping our resource base (raw materials) in response to the changing business environment. |  |  |  |  |  |

| **Adaptability in processes** | | | | | |
| --- | --- | --- | --- | --- | --- |
| We continually strive to further reduce delivery times for our products or services through more efficient operations. |  |  |  |  |  |
| We stand out for taking advantage of changes or interruptions in the market. |  |  |  |  |  |
| We develop innovative technologies to improve our operations. |  |  |  |  |  |
| We effectively employ continuous improvement programs. |  |  |  |  |  |

**Definition of Resilience**: “The adaptive capability of a firm’s supply chain to prepare for unexpected events, respond to disruptions, and recover from them in a timely manner by maintaining continuity of operations at the desired level of connectedness and control over structure and function”.

| **Resilience** | | | | | |
| --- | --- | --- | --- | --- | --- |
| On a scale from 1 to 5, where 1 is strongly disagree and 5 is strongly agree, please rate the following statements.  Consider your relationships with both suppliers and customer.  Please, rate your level of agreement on the following resilience related statements: | **Strongly disagree** | **Disagree** | **Neither agree nor disagree** | **I agree** | **Completely agree** |
|  | **1** | **2** | **3** | **4** | **5** |
| Agility is fundamental to rapidly recover from unwanted disruptions |  |  |  |  |  |
| High flexibility is required to cope with unplanned failures |  |  |  |  |  |
| There is a need for risk and revenue sharing strategy communicated to all employees |  |  |  |  |  |
| All business partners in the supply chain should share information for business purposes |  |  |  |  |  |
| Business partners associated with the supply chain collaborate for reducing the risks in supply chain operations |  |  |  |  |  |
| There is a need to assess information security related risks in the supply chain business processes |  |  |  |  |  |
| Visibility is important across the supply chain to anticipate and respond to unexpected events |  |  |  |  |  |
| There is a need for adaptive capability to build up resilience across the supply chain |  |  |  |  |  |
| Trust should be required between employees, contractors and third party users depending on the information to be accessed |  |  |  |  |  |
| It is necessary to have a focus on supply chain structure for reducing the risk arising due to disruption in supply chain operations |  |  |  |  |  |
| Supply chain resilience is an important aspect for achieving sustainability |  |  |  |  |  |
| It is useful to have a strategic risk planning for supply chain resilience related activities |  |  |  |  |  |
| It is important to share risk management knowledge between supply chain partners |  |  |  |  |  |
| Assets redundancy (i.e. extra Inventory, fleet, additional capacity) ensures continuity of operations |  |  |  |  |  |
| The access to financial assets adds to the recovery of unexpected events |  |  |  |  |  |
| Detailed scenario planning is useful to minimize the cost of disruptive events |  |  |  |  |  |
